# Contact Toxicity and Repellency of the Essential Oil of *Liriope muscari* (DECN.) Bailey against Three Insect Tobacco Storage Pests

**DOI:** 10.3390/molecules20011676

**Published:** 2015-01-20

**Authors:** Yan Wu, Wen-Juan Zhang, Ping-Juan Wang, Kai Yang, Dong-Ye Huang, Jian-Yu Wei, Zhao-Fu Tian, Jia-Feng Bai, Shu-Shan Du

**Affiliations:** 1 Technical Center of China Tobacco Guangxi Industrial Co., Ltd, Nanning 530001, Guangxi, China; E-Mails: abelabel@126.com (Y.W.); 15177471944@163.com (P.-J.W.); huangdongye8@163.com (D.-Y.H.); jtx_wjy@163.com (J.-Y.W.); tianzhaofu@yeah.net (Z.-F.T.); baijiafeng10@163.com (J.-F.B.); 2 Beijing Key Laboratory of Traditional Chinese Medicine Protection and Utilization, Beijing Normal University, No.19 Xinjiekouwai Street, Beijing 100875, China; E-Mails: zwj0729@mail.bnu.edu.cn (W.-J.Z.); yangk_1988@mail.bnu.edu.cn (K.Y.)

**Keywords:** *Liriope muscari*, contact activity, repellency, essential oil composition, stored product insects

## Abstract

In order to find and develop new botanical pesticides against tobacco storage pests, bioactivity screening was performed. The essential oil obtained from the aerial parts of *Liriope muscari* was investigated by GC/MS and GC/FID. A total of 14 components representing 96.12% of the oil were identified and the main compounds in the oil were found to be methyl eugenol (42.15%) and safrole (17.15%), followed by myristicin (14.18%) and 3,5-dimethoxytoluene (10.60%). After screening, the essential oil exhibit potential insecticidal activity. In the progress of assay, it showed that the essential oil exhibited potent contact toxicity against *Tribolium castaneum*, *Lasioderma serricorne* and *Liposcelis bostrychophila* adults, with LD_50_ values of 13.36, 11.28 µg/adult and 21.37 µg/cm^2^, respectively. The essential oil also exhibited strong repellency against the three stored product insects. At the same concentrations, the essential oil was more repellent to *T. castaneum* than to *L. serricorne* adults. The results indicate that the essential oil of *Liriope muscari* has potential to be developed into a natural insecticide or repellent for controlling insects in stored tobacco and traditional Chinese medicinal materials.

## 1. Introduction

With the rapid development of the tobacco industry in China, the size and number of tobacco storage facilities is becoming larger and larger. However, it is very easy for tobacco leaves to be effected by storage insects [1]. Coleopterans have global importance in grain storage and industrial food production and can cause damage to the appearance of products as well as weight loss and reduced nutrient levels, rendering products unfit for human consumption [2]. The red flour beetle (*Tribolium castaneum* Herbst) (Coleoptera: Tenebrionidae) is distributed world-wide and among the most economically important stored product pests [3]. The cigarette beetle (*Lasioderma serricorne* Frbricius) (Coleoptera: Anobiidae) is a widespread and destructive primary insect pests of stored cereals, tobacco, oilseeds, dried fruits and traditional Chinese medicinal materials [4]. The booklouse (*Liposcelis bostrychophila* Badonnel) (Psocoptera) is frequently found in stored product grains and traditional medicines, or other amylaceous products [5]. At present, phosphine is the most widely used fumigant, but threatens human health [6]. In recent years, research and development on botanical pesticides have attracted great attention in the whole world. Volatile plant ingredients which are highly selective, easy to decompose, leave no residue, and cause no pollution or threats to human and environmental harmony, such as plant essential oils, are found in botanical pesticides [7,8]. Botanical pesticides have the advantage of providing novel modes of action against insects that can reduce the risk of cross-resistance as well as offering new leads for the design of target-specific molecules. In previous studies, the bioactivities of a large number of essential oils have been also evaluated against a number of stored product insects [9,10,11,12].

The species *Liriope muscari* (Decne.) Bailey is a perennial herb, belonging to the Liriope of the Liliaceae family, and native to East Asia, including China, Japanese and North Korea. The dried tuberous roots have been used as the medicinal part of this plant to treat cough, insomnia, astriction and other diseases [13]. Genuine medicinal materials containing this material are mainly distributed in Quanzhou and Putian, Fujian Province [14]. A literature survey has shown that *L. muscari* has anti-tumor metastatic activity [15], immune functions [16,17], anti-inflammatory [18], anti-thrombotic activity [19], antioxidant activity [20] and nematocidal activity [14]. The chemical constituents of the root tuber of *L. muscari* have been reported [14,20,21,22,23,24]. However, there is no report on the volatile constituents and bioactivity of the essential oil of *L. muscari* aerial parts. Here, the chemical constituents and bioactivities of the essential oil of *L. muscari* aerial parts were investigated against three stored product insects for the first time.

## 2. Results and Discussion

### 2.1. Chemical Composition of the Essential Oil

The chemical composition of the essential oil of *L. muscari* aerial parts is reported here for the first time. The essential oil yield of *L. muscari* was 0.05% (v/w) and the density of the concentrated essential oil was determined as 1.18 g/cm^3^. The GC/MS and GC/FID analysis results for the oil are presented in Table 1. The analyses revealed 14 components, representing 96.12% of the oil. The main components were methyl eugenol (42.15%), safrole (17.15%), myristicin (14.18%), 3,5-dimethoxytoluene (10.60%) and 3,4,5-trimethoxytoluene (9.05%).

**Table 1:** Chemical composition of *Liriope muscari* essential oil.

Peak No.	Compound	RI *	Relative Content (%)
**1**	Linalool	1075	0.18
**2**	Camphor	1143	0.05
**3**	Eucarvone	1146	0.38
**4**	Borneol	1156	0.33
**5**	α-Terpineol	1181	0.31
**6**	Estragole	1195	0.28
**7**	3,5-Dimethoxytoluene	1264	10.60
**8**	Safrole	1285	17.15
**9**	Linalool propionate	1340	0.05
**10**	Methyleugenol	1403	42.15
**11**	3,4,5-Trimethoxytoluene	1408	9.05
**12**	Germacrene D	1476	0.11
**13**	Myristicin	1523	14.18
**14**	Elemicin	1554	1.30
	Phenylpropanoids		75.06
	Monoterpenoids		1.30
	Sesquiterpenoids		0.11
	Aromatic compounds		19.65
	Total		96.12

* RI, retention index as determined on a HP-5MS column using the homologous series of *n*-alkanes.

### 2.2. Contact Toxicity

The crude essential oil of *L. muscari* aerial parts showed pronounced contact toxicity against *T. castaneum*, *L. serricorne* and *L. bostrychophila* with LD_50_ values of 13.36, 11.28 µg/adult and 21.37 µg/cm^2^, respectively (Table 2).

**Table 2:** Contact toxicity of *Liriope muscari* essential oil against *Tribolium castaneum* (TC), *Lasioderma serricorne* (LS) and *Liposcelis bostrychophila* (LB) adults.

Insect	Compounds	LD_50_ *	95% FL *	Slope ± SE	Chi square (χ^2^)
LS	Essential oil	11.28	10.22–12.43	4.59 ± 0.48	16.10
	Pyrethrins **	0.24	0.16–0.35	1.31 ± 0.20	17.36
TC	Essential oil	13.36	10.94–15.70	1.67 ± 0.26	11.96
	Pyrethrins	0.26	0.22–0.30	3.34 ± 0.32	13.11
LB	Essential oil	21.37	20.17–22.45	8.60 ± 1.01	10.35
	Pyrethrins **	18.72	17.60–19.92	2.98 ± 0.40	10.56

* Concentration (µg/adult)/(µg/cm^2^); ** data from Yang *et al.* [25].

According to the data listed in Table 2, the essential oil of *L. muscari* aerial parts exhibited stronger contact toxicity against *L. bostrychophila* than *T. castaneum* and *L. serricorne.* When compared with the positive control, pyrethrins, the essential oil demonstrated 51, 47 and 1.1 times less toxicity against *T. castaneum*, *L. serricorne* adults and *L. bostrychophila*, respectively. This showed that *L. bostrychophila* were most susceptible to the contact toxicity of the essential oil of *L. muscari* and *T. castaneum* adults were more tolerant than *L. serricorne* adults. However, compared with other essential oils mentioned in the literature, the essential oil of *L. muscari* aerial parts possessed stronger contact toxicity against *T. castaneum* adults, e.g., essential oils of *Dracocephalum moldavica* (LD_50_ = 18.28 µg/adult) [26], *Murraya exotica* (LD_50_ = 20.94 µg/adult) [20], *Evodia lepta* (LD_50_ = 166.94 µg/adult) [22]. The essential oil of *L. muscari* aerial parts also exhibited stronger contact toxicity against *L. bostrychophila* than other reported oils, for example essential oils of *Lonicera japonica* (LD_50_ = 64.04 µg/cm^2^) [5], *Foeniculum vulgare* (LD_50_ = 90.36 µg/cm^2^) [27], *Acorus calamus* (LD_50_ = 100.21 µg/cm^2^) [28], *Curcuma wenyujin* (LD_50_ = 208.85 µg/cm^2^) [29].

In another reference [30], six compounds, including, eugenol, methyl eugenol, methyl isoeugenol, elemicin, myristicin and safrole isolated from *Myristica fragrans* (LD_50_ = 19.3 µg/adult) showed potent contact toxicity against *L. serricorne* adults with LD_50_ values of 13.2, 12.8, 21.3, 9.8, 20.5 and 14.6 µg/adult, respectively. Methyl eugenol, safrole and myristicin are also the main compounds of the essential oil of *L. muscari*, hence, the revealed toxic properties of the essential oil of *L. muscari* on those three tobacco storage insects might be attributed to the synergistic effects of its diverse major and minor components. Also, the different volatility of oils and different mechanisms of action on different insects might lead to different contact toxicity. However, there is insufficient data at present to conform these hypotheses. In addition, further investigations need to be conducted in the future to determine whether the oil is safe to humans or not.

### 2.3. Repellency

In addition to contact activities, the repellent effect of the essential oil of *L. muscari* aerial parts against the three stored product insects was also investigated. The results are presented in Table 3 and Table 4.

**Table 3:** Pecentage repellency (PR) after two exposure times for the essential oil against *Tribolium castaneum* (TC) and *Lasioderma serricorne* (LS) adults ^a^.

Insect	Treatment	2 h/4 h				
		78.63 *	15.73 *	3.15 *	0.63 *	0.13 *
TC	Crude oil	84 ± 3a; 82 ± 5a	92 ± 5a; 90 ± 4a	84 ± 7a; 82 ± 10a	74 ± 10a; 68 ± 5a	72 ± 8a; 66 ± 7a
	DEET	100 ± 0a; 94 ± 6a	96 ± 3a; 80 ± 10a	74 ± 16a; 46 ± 17b	66 ± 9a; −2 ± 20b	18 ± 16b; −8 ± 22b
LS	Crude oil	86 ± 6a; 82 ± 3a	68 ± 9a; 52 ± 8a	18 ± 8a; 30 ± 10a	6 ± 17a; 8 ± 12a	8 ± 21a; −12 ± 20a
	DEET	88 ± 7a; 98 ± 4a	76 ± 14a; 78 ± 9b	28 ± 7a; 58 ± 16b	20 ± 14b; 56 ± 14b	16 ± 7a; 46 ± 7b

* Concentration (nL/cm^2^); ^a^ Means in the same column followed by the same letters do not differ significantly (*p* < 0.05) in ANOVA and Tukey’s tests. PR was subjected to an arcsine square-root transformation before ANOVA and Tukey’s tests.

The essential oil strongly repelled all tested species of stored product insects, but the repellent effect was more marked on *T. castaneum* than on *L. serricorne*. Data showed that at the tested concentration of 0.13 nL/cm^2^, the crude oil still showed strong repellency (class IV) against the red flour beetle, *T. castaneum* adults at 2 h, 4 h after exposure (Table 3) while no repellency of the positive control, DEET, was seen, but some insect attractant properties were observed. Moreover, at the assayed concentrations of 78.63, 15.73 and 3.15 nL/cm^2^, the essential oil exhibited strong repellency (class V) at 2 h, 4 h after exposure (Table 3). 

**Table 4:** Percentage repellency (PR) after two exposure times for the essential oil against *Liposcelis bostrychophila* (LB) ^a^.

Insect	Treatment	2 h/4 h				
		31.58 *	6.32 *	1.26 *	0.25 *	0.05 *
LB	Crude oil	80 ± 10a; 78 ± 5a	100 ± 0a; 96 ± 3a	92 ± 3a; 86 ± 3a	50 ± 13a; 42 ± 19a	24 ± 7a; 16 ± 6a
	DEET	100 ± 0a; 96 ± 7b	92 ± 7a; 90 ± 10a	92 ± 7a; 90 ± 6a	74 ± 9b; 72 ± 19b	30 ± 10a; 32 ± 7b

* Concentration (nL/cm^2^); ^a^ Means in the same column followed by the same letters do not differ significantly (*p* < 0.05) in ANOVA and Tukey’s tests. PR was subjected to an arcsine square-root transformation before ANOVA and Tukey’s tests.

The repellency against *L. serricorne* is weaker than against *T. castaneum*. Compared with the positive control, DEET, only at the highest tested concentration of 78.63 nL/cm^2^, the essential oil of *L. muscari* possessed the same level and strong repellency (class V) against *L. serricorne* adults (Table 3). As the concentrations were reduced, the repellency of the crude oil against *L. serricorne* also tended to drop off. Moreover, at the lowest concentration, no repellency of the crude oil, but some insect attractant properties were observed at 4 h after exposure (Table 3).

At the same time, the essential oil of *L. muscari* also showed strong repellency against the booklouse, *L. bostrychophila*. At the tested concentration of 0.25 nL/cm^2^, the crude oil still exhibited strong repellency (class III) at 2 h, 4 h after exposure, while at the assayed concentrations of 6.32, 1.26 nL/cm^2^, the oil showed strongly repellent against booklice at 2 h, 4 h after exposure (Table 4). However, at the lowest concentration, the repellency of the oil against booklice was weaker compared with the positive control, DEET (Table 4). Many essential oils have been evaluated for repellency against insects [31]. In China, essential oils derived from spices and Chinese medicinal herbs were also evaluated for insecticidal activity and repellency against insects [28,29,32,33,34]. In this article, we report the contact and repellent activities of the essential oil of *L. muscari* aerial parts for the first time. Above all, the essential oil of *L. muscari* possessed strong repellency against the three stored product insects. These findings, considered together, suggest that the essential oil of *L. muscari* shows potential for development as a natural repellent for stored products.

## 3. Experimental Section

### 3.1. Plant Material and Extractions

Fresh aerial parts (2 kg) of *Liriope muscari* were harvested in 2013 from Quanzhou (Fujian Province, China, N latitude: 24°30'–25°56'; E longitude: 117°25'–119°05'). The plant species was identified and a voucher specimen (BNU-dushushan-2013-05-24-06) was deposited at the Herbarium of the College of Resources Science and Technology, Beijing Normal University (Beijing, China). The aerial parts of *Liriope muscari* were air dried for 1 week and ground to a powder. The ground powder of *L. muscari* was subjected to hydrodistillation using a modified Clevenger-type apparatus for 8 h and extracted with *n*-hexane. Anhydrous sodium sulphate was used to remove water after extraction. The essential oil was stored in an airtight container in a refrigerator at 4 °C.

### 3.2. Insects

Red flour beetles (*T. castaneum*), cigarette beetles (*L. serricorne*) and booklice (*L. bostrychophila*) were obtained from laboratory cultures maintained in the dark in incubators at 29–30 °C and 70%–80% relative humidity (RH). The red flour beetles and cigarette beetles were reared on wheat flour mixed with yeast (10:1, w/w) at 12%–13% moisture content while the booklice were reared on a 1:1:1 mixture, by mass, of milk powder, active yeast and flour. The unsexed adult beetles/booklice used in all the experiments were about 1–2 weeks old. All containers housing insects and the Petri dishes used in experiments were made escape proof with a coating of polytetrafluoroethylene (Fluon).

### 3.3. Contact Toxicity

The contact toxicity of the essential oil of *L. muscari* aerial parts against *T. castaneum* and *L. serricorne* adults was measured by using topical application as described by Liu and Ho [35]. Serial dilutions of essential oil (10%–1.97% (v/v) for *T. castaneum* and *L. serricorne*; five concentrations) were prepared in *n*-hexane. Preliminary experiments were conducted to determine appropriate ranges of test concentrations. Aliquots (0.5 µL) of the dilutions were applied topically to the dorsal thorax of the insects. Controls were established using *n*-hexane. Both treated and control insects were then transferred to glass vials (ten insects per vial) with culture media and kept in incubators at 29–30 °C and 70%–80% RH. Mortality of insects was observed daily after treatment. The experiments were repeated three times. While the contact toxicity of the essential oil against *L. bostrychophila* was also tested as described [5]. A 5.5 cm diameter filter paper was treated with 300 µL of the solution of the essential oil. The filter paper after treated with solid glue was placed in a 5.5 cm diameter Petri dish and 10 booklice were put on the filter paper. A cover was put and all the Petri dishes were kept in incubators. *n*-Hexane was used as a negative control. Five concentrations (in *n*-hexane) and five replicates of each concentration were used. As a positive control, pyrethrins (pyrethrin 1: 24%; pyrethrin 2: 13%; cinnerin 1: 2%; cinnerin 2: 2%; jasmolin 1: 1%; jasmolin 2: 1%) was used under the conditions as the oil. Mortality of insects was observed after 24 h. The LD_50_ values were calculated by using Probit analysis [36].

### 3.4. Repellency

The repellent effects of the essential oil against *T. castaneum*, *L. serricorne* and *L. bostrychophila* were assessed by using assays on Petri dishes [37]. Petri dishes 9 cm in diameter were used to confine beetles during the experiment for *T. castaneum* and *L. serricorne*. The essential oil of *L. muscari* aerial parts was prepared in *n*-hexane (78.63, 15.73, 3.15, 0.63 and 0.13 nL/cm^2^), and absolute *n*-hexane was used as the control. Filter paper 9 cm in diameter was cut in half and 500 µL of each concentration was applied separately to half of the filter paper as uniformly as possible with a micropipette. The other half (control) was treated with 500 µL of absolute *n*-hexane. Both the treated half and the control half were then air-dried to evaporate the solvent completely (30 s). A full disk was carefully remade by attaching the tested half to the negative control half with tape. Each reassembled filter paper after treatment with solid glue was placed in a Petri dish with the seam oriented in one of four randomly selected different directions to avoid any insecticidal stimuli affecting the distribution of insects. Twenty insects were released in the center of each filter paper disk, and a cover was placed over the Petri dish. Five replicates were used, and the experiment was repeated three times. Counts of the insects present on each strip were made after 2 and 4 h. The percent repellency (PR) of each volatile oil was then calculated using the formula

PR (%)=[(Nc−Nt)/(Nc+Nt)]×100

where *Nc* is the number of insects present in the negative control half and *Nt* is the number of insects present in the treated half. The averages were then assigned to different classes (0 to V) using the following scale (percentage repellency) [32]. Class, % repellency: 0, >0.01 to <0.1; I, 0.1–20.0; II, 20.1–40.0; III, 40.1–60.0; IV, 60.1–80.0 and V, 80.1–100.

As for the booklice, Petri dishes and filter papers were changed to 6 cm in diameter and the concentration of the oil used in the experiments were 31.58, 6.32, 1.26, 0.25, 0.05 nL/cm^2^. The half filter paper was treated with 150 µL of the solution. As a positive control, a commercial repellent DEET (*N*,*N*-diethyl-3-methylbenzamide), was used under the conditions as the oil. Analysis of variance (ANOVA) and Tukey’s test were conducted by using SPSS Statistics ver. 20 for Windows 2007. Percentage was subjected to an arcsine square-root transformation before ANOVA and Tukey’s tests.

### 3.5. GC/MS and GC/FID Analysis

Components of the essential oil of *L. muscari* were separated and identified using a Bruker-320 gas chromatography/mass spectrometry (GC/MS, Bruker Daltonics Inc., Billerica, MA, USA) system equipped with a flame ionization detector (GC-FID, Bruker Daltonics Inc.) and a HP-5MS (30 m × 0.25 mm × 0.25 µm, Bruker Daltonics Inc.) capillary column. The GC settings were as follows: the initial oven temperature was held at 50 °C for 4 min and ramped at 10 °C·min^−1^ to 290 °C for 17 min. The injector temperature was maintained at 250 °C. The samples (1 µL, diluted to 1% with hexane) were injected, with a split ratio of 1:60. The carrier gas was helium at flow rate of 1 mL/min. Spectra were scanned from 45 to 650 *m/z* at 2 scans/s. Most constituents were identified by gas chromatography by comparison of their retention indices with those of the literature or with those of authentic compounds available in our laboratories. The retention indices were determined in relation to a homologous series of *n*-alkanes (C_5_–C_35_) under the same operating conditions. Further identification was made by comparison of their mass spectra with those stored in NIST 05 (Standard Reference Data, Gaithersburg, MD, USA) and Wiley 275 libraries (Wiley, New York, NY, USA) or with mass spectra from literature [38]. Component relative percentages were calculated based on GC peak areas, without using correction factors.

## 4. Conclusions

The composition of the essential oil of *L. muscari* aerial parts and the contact toxicity and repellency of the essential oil against three stored product insects for the first time. The study indicates that the essential oil of *L. muscari* aerial parts has potential for development into a natural insecticide/repellent for the control of insects in stored products. 
